# Variable-interval temporal feathering to optimize organs-at-risk repair for head and neck adaptive radiotherapy

**DOI:** 10.1016/j.phro.2026.101026

**Published:** 2026-06-19

**Authors:** Aysenur Karagoz, Mehdi Hemmati, Fatemeh Nosrat, Panayiotis Mavroidis, Cem Dede, Lucas B. McCullum, Raul Garcia, Seyedmohammadhossein Hosseinian, Jacob G. Scott, James E. Bates, Heiko Enderling, Abdallah S.R. Mohamed, Kristy K. Brock, Andrew J. Schaefer, Clifton D. Fuller

**Affiliations:** a Department of Computational Applied Mathematics and Operations Research, Rice University, Houston, TX, United States of America; b Industrial Engineering Department, Bilkent University, Ankara, Turkey; c School of Industrial and Systems Engineering, University of Oklahoma, Norman, OK, United States of America; d Stephenson Cancer Center, University of Oklahoma Health Science Center, Norman, OK, United States of America; e Department of Industrial and Systems Engineering, University of Florida, Gainesville, FL, United States of America; f Department of Radiation Oncology, School of Medicine, University of North Carolina, Chapel Hill, NC, United States of America; g Department of Radiation Oncology, Division of Radiation Oncology, University of Texas MD Anderson Cancer Center, Houston, TX, United States of America; h Edward P. Fitts Department of Industrial and Systems Engineering, North Carolina State University, Raleigh, NC, United States of America; i Department of Translational Hematology and Oncology Research, Cleveland Clinic, Cleveland, OH, United States of America; j Department of Radiation Oncology, School of Medicine, Emory University, Atlanta, GA, United States of America; k Department of Radiation Oncology, Baylor College of Medicine, Houston, TX, United States of America; l Department of Imaging Physics, Division of Diagnostic Imaging, University of Texas MD Anderson Cancer Center, Houston, TX, United States of America; m Department of Radiation Physics, Division of Radiation Oncology, University of Texas MD Anderson Cancer Center, Houston, TX, United States of America

**Keywords:** head-and-neck cancer radiotherapy, variable daily dose, temporally feathered radiation therapy, mixed-integer programming

## Abstract

*Background and Purpose:* Temporally feathered radiation therapy (TFRT) for head-and-neck cancer (HNC) combines variable-dose intensity modulated radiation therapy (IMRT) subplans to increase organs-at-risk's (OAR) recovery. This study evaluated whether incorporating OAR-specific radiosensitivity into variable rest-time planning can reduce side effects.

*Materials and Methods:* Using radiosensitivity profiles of five OARs (larynx, esophagus, parotid, spinal cord, brainstem), we developed an optimized TFRT (oTFRT) model to maximize OAR recovery by optimizing rest times, guided by the linear-quadratic and normal tissue complication probability models. High-dose sparing factors were generated using Monte Carlo simulation. OAR recovery under IMRT and standard TFRT (sTFRT) was compared with oTFRT in two experiments: one with total oTFRT dose matched to IMRT, and one without dose restrictions.

*Results:* Under dose-matched experiment, the oTFRT yielded improved recovery for larynx (88.1% versus 75.9%), parotid (67.5% versus 58.5%), and esophagus (93.6% versus 89.1%), while brainstem recovery (83.9% versus 82.4%) and spinal cord recovery (67.5% versus 65.0%) were slightly higher than IMRT. Under dose-unrestricted experiment, oTFRT improved recovery for brainstem and spinal cord (88.5% and 77.9%, respectively) versus the second-best plan (83.9% and 66.9%), while parotid recovery was slightly higher (67.2% versus 66.7%). The larynx showed improved recovery relative to IMRT (81.7% versus 75.9%) but remained below sTFRT (88.0%), whereas esophagus recovery was lower (84.8% versus 93.6% for sTFRT). Despite delivering higher doses to larynx and esophagus, oTFRT maintained comparable or improved recovery relative to IMRT.

*Conclusions:* By considering nonuniform OARs' radiosensitivity, oTFRT can improve recovery and potentially reduce side effects, warranting further consideration.

## Highlights

• Reducing side effects in head-and-neck radiotherapy via temporal feathering.
• Optimization model deciding nonuniform organ rest times based on radiosensitivity.
• Compared to the standard treatment plan, recovery improved up to 10.6%.

## Graphical abstract

**Figure:**
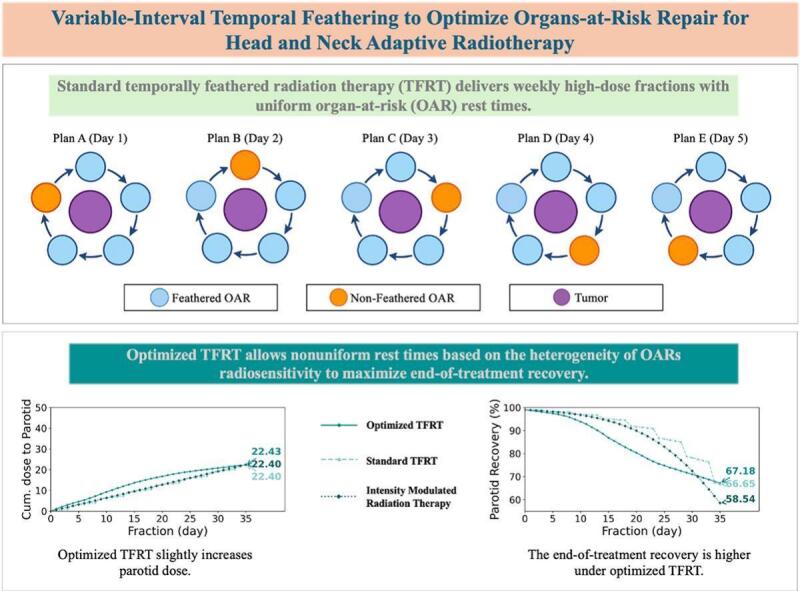

## 1 Introduction

According to the latest “Global cancer statistics 2022: GLOBOCAN” estimates, head-and-neck cancer (HNC) represents a global burden with more than 680,000 new cases diagnosed each year and approximately 344,000 deaths worldwide [1]. Intensity-modulated radiation therapy (IMRT) in combination with cisplatin chemotherapy remains the primary curative treatment approach for HNC patients [2], [3], [4]. IMRT has achieved successful oncological results with increased locoregional control and survival in HNC patients compared to the conventional RT [5]. With the rise in human papillomavirus (HPV)-related cases, which have a better prognosis compared to HPV-negative cases (80% versus 40% five-year survival [6]), the five-year overall survival rate of HNC is now 68.5% [7].

Most HNC patients treated with IMRT experience reduced quality of life from radiation-induced side effects, such as xerostomia and dysphagia, which stem directly from the injury to nearby organs-at-risk (OARs) [8]. With improved survival rates and the focal shift of radiation therapy (RT) treatment planning toward sparing normal tissues, improving the quality of life in HNC survivors has now become a primary goal [9].

In a standard IMRT plan for HNC, a fixed daily tumor dose (∼2 Gy) is delivered using identical treatment plan each fraction, resulting in daily uniform dose distributions to surrounding OARs. However, recent studies report potential benefits of variable fraction dose based on the non-homogeneity in tumor cells and late-treatment effects such as increased tumor repopulation rate [10]. Nonuniform fractionation can also arise in adaptive radiation therapy (ART) in which treatment plans are modified during the course of therapy to account for anatomical changes, which can subsequently result in variations in daily dose distributions. However, most studies focus on nonuniform hypofractionating subregions of the tumor while delivering a fixed daily dose to OARs [11], [12].

Alfonso et al. [13] proposed temporally feathered RT (TFRT), which allows variable daily doses to OARs to improve sublethal damage repair. A TFRT plan is constructed as a composite of several isocurative subplans, each delivered on a specific day. For HNC patients, five OARs are typically considered [13]. Each day, an OAR (termed as the “temporally feathered” OAR) receives a slightly higher radiation dose compared to the fixed dose prescribed by a standard IMRT plan (but within the maximum dose threshold to avoid lethal damage), allowing reduced radiation to all other OARs. Therefore, each OAR receives a higher-than-standard fractional dose of IMRT plan once weekly, with lower doses in the remaining four days. The hypothesis is that the one-week interval between the higher fractional doses will promote sublethal damage repair and repopulation, which are shown to take weeks to months to occur after the peak acute side effects [14]. Simulated and in vivo studies suggest TFRT can reduce side effects in HNC patients when the higher-than-standard doses are carefully chosen [15], [16].

Existing TFRT studies employ a fixed (one-week) recovery interval between consecutive high-dose fractions delivered to the same OAR [13], [15], [16], [17]. For example, Arnold et al. [17] recently proposed a direct aperture optimization framework that jointly optimizes multiple TFRT subplans within a single treatment course while maintaining this fixed interval structure. From the radiobiological standpoint, the use of a common recovery interval across all OARs implicitly assumes similar radiation response and repair characteristics among OARs in HNC RT. However, as predicted by the linear-quadratic (LQ) model [18], the recovery time among OARs may vary as they often exhibit different levels of radiosensitivity, thus some OARs may have higher recovery rates while others require longer rest time. For example, the brainstem and spinal cord exhibit significantly different levels of radiosensitivity with the former being more sensitive to radiation dose, thus gaining relatively higher benefit from fractionation [19]. While existing works in HNC TFRT assume identical radiosensitivity parameters across all OARs, a critical gap remains in understanding whether incorporating OAR-specific radiosensitivity to allow nonuniform recovery times between high-dose fractions can further reduce treatment-related side effects.

In this work, we aimed to present the concept of a variable schedule for OAR feathering by investigating the effect of variable radiosensitivity profiles across OARs on the outcomes of TFRT. Specifically, we developed a mathematical optimization model that allows variable recovery times between high-dose days by computing the optimal “OAR feathering” scheme for TFRT treatment planning. Further, we leveraged our model to study the outcomes of TFRT in the presence of specific organ-preserving goals, which can be set according to the clinician's preferences.

## 2 Materials and methods

### 2.1 Mathematical optimization model

Our model integrated the LQ model [18] (Supplementary Material A) and a non-spatial dynamical tissue-response model [20] (Supplementary Material B). The LQ model predicted cell survival based on dose and radiosensitivity parameters (α,β), with the α/β ratio reflecting fractionation sensitivity [21]. The dynamical model functioned as an NTCP framework to estimate radiation-induced OAR damage. While both mechanistic [22], [23], [24], [25], [26] and data-driven [27], [28], [29], [30] NTCP models exist, we adopted the model of [20], which had previously been applied to evaluate TFRT [13]. Through developing a mixed-integer programming model, we computed optimal feathering schedules for the OARs to maximize the weighed end-of-treatment OAR recovery levels subject to a set of constraints discussed next.

To account for differences in tissue response, OAR-specific radiosensitivity parameters were incorporated into the optimization model. These parameters were used to select the daily subplan to maximize (weighted) end-of-treatment OAR recovery. Constraints were applied using the concept of sparing factors [31], [32], [33] to compute OAR dose based on tumor dose. Higher (lower) sparing factors were used to model high-dose (low-dose) days as stated by Alfonso et al. [13]. This resulted in OAR doses within the 0.5–2.5 Gy range reported in that study and served to evaluate the effects of temporal dose redistribution in a proof-of-concept framework, while remaining close to the original TFRT study. We also included constraints requiring exactly one OAR is feathered per fraction (Supplementary Material C).

Although TFRT allows a slightly higher dose to one OAR each day, the cumulative biologically effective dose (BED) to each OAR must remain within acceptable limits. Therefore, maximum BED constraints were included to prevent excessive dose accumulation (Supplementary Material D). Additional constraints were used to model OAR recovery over time by incorporating the discretized ordinary differential equation in [20]. The recovery level of each OAR also guided daily subplan selection through a minimum recovery threshold required before an OAR could be scheduled for a “high-dose” day (Supplementary Material E).

Two experimental settings were considered to evaluate optimized TFRT schedules. In the dose-matched TFRT experiment, the cumulative dose delivered to each OAR was constrained to be at least equal to that delivered under the corresponding IMRT plan (Supplementary Material F). In the dose-unrestricted TFRT experiment, no cumulative OAR dose constraint was imposed, consistent with the original TFRT study [13]. The resulting optimized TFRT schedules were compared with the corresponding IMRT plans under both settings.

The integrated model maximized the weighted sum of OAR's end-of-treatment recovery, with weights reflecting clinician's preference for sparing certain OARs, subject to the constraints described above (Supplementary Material G).

### 2.2 Model input parameters for OAR feathering

The feathered OARs (brainstem, larynx, esophagus, parotid, and spinal cord) considered in this study differ from those in Parsai et al. [15] due to the lack of available radiobiological parameters, and the brainstem was included based on clinical recommendations. Alfonso et al. [13] apply a fixed α/β ratio of 3 Gy across all OARs. Our radiosensitivity parameters were based on the values of Kehwar [19], where α and β range between 0.04 and 0.08 and 0.01–0.02, respectively. Since the original TFRT study [13] used significantly higher $\left(\alpha ,\beta \right)$ values, we scaled the values reported in [19] to align in magnitude with those of the original TFRT study [13]. Our values were consistent with the literature (varying between 2 and 4 Gy for late-responding OARs). We applied a universal recovery threshold, ξ=0.75. This choice was motivated by two considerations: (1) in our NTCP framework, each OAR's dose response was modeled as a sigmoid function, with the 0.75 threshold located in the steep transition region where recovery remained likely but unsaturated; and (2) clinically, the shift from Grade 1 (mild) to Grade 2 (moderate) side effects often corresponded to a 20–30% decline in objective physiological measures. For the recovery rate μ of the NTCP model (Supplementary Materials B and E), we chose a value of 0.05 based on the reported doubling time of 14 days by Stocks et al. [34], leading to a recovery rate of $\mu =\ln\left(2\right)/14=0.05$.

The maximum allowable BED values for OARs were computed based on [35], [36] (Table 1). On low-dose days, all OARs receive the same dose across subplans; therefore, each subplan is fully characterized by a high-dose sparing factor and a corresponding low-dose sparing factor (Table 2). Sparing factors were derived from the IMRT plan following the TFRT framework in [15], where the base sparing factor is defined as the ratio of the fixed daily dose delivered to the OARs and the tumor (2 Gy per fraction). To model variability, high-dose day sparing factors were generated using Monte Carlo simulation with 100 samples. Alfonso et al. [13] shows that OARs benefit when the difference between the high-dose and standard dose (ΔH) lies between 1.8 and 2 Gy, with a corresponding low-dose reduction ΔL = 0.4 Gy. Accordingly, high-dose sparing factors (SF_high) were sampled uniformly from 0.9 to 1, consistent with this regime under a 2 Gy tumor dose per fraction. Sparing factors greater than 1 were not considered, as they would imply delivering a higher dose to the OAR than to the tumor. The sparing factors for low-dose days (SF_low) were computed to preserve total dose equivalence with the total dose received by the OAR under the reference IMRT plan. Assuming a constant tumor dose of 2 Gy per fraction over 35 fractions, the low-dose sparing factor was calculated using Eq. (1).

(1) $$\mathrm{SF}\_\mathrm{low}=\text{total IMRT dose}-7\times \left(2\times \mathrm{SF}\_\text{high}\right))/\left(28\times 2\right)$$

**Table 1:** Radiosensitivity parameters (*α,β*) recovery rate (μ), recovery threshold (ζ)†, and maximum BED allowed to the organs-at-risk (OAR) (τ).

**OARs**	*α***(Gy**^**−1**^**)** [13], [19]	*β***(Gy**^**−2**^**)** [13], [19]	μ**(day**^**−1**^**)** [34]	ξ^†^	τ**(Gy)** [35], [36]
Brainstem	0.1964	0.0936	0.05	0.75	95
Larynx	0.3252	0.0836	0.05	0.75	110
Esophagus	0.2832	0.0944	0.05	0.75	92
Parotid	0.2512	0.0836	0.05	0.75	117
Spinal Cord	0.178	0.054	0.05	0.75	104

† Explained in Section 2.2.

**Table 2:** Sparing factors for the experiments were computed using the daily dose received by the organs [15], [37]. OAR: organs at risk; IMRT: intensity modulated radiation therapy.

OARs	High-Dose Sparing Factor (Mean ± SD)	Low-Dose Sparing Factor (Mean ± SD)	IMRT Sparing Factor	IMRT Total Dose (Gy)
Brainstem	0.9579 ± 0.0281	0.1355 ± 0.0070	0.300	21.00 [37]
Larynx	0.9517 ± 0.0299	0.0433 ± 0.0075	0.225	15.75 [15]
Esophagus	0.9471 ± 0.0272	0.0195 ± 0.0068	0.205	14.35 [15]
Parotid	0.9525 ± 0.0291	0.1619 ± 0.0073	0.320	22.40 [15]
Spinal Cord	0.9504 ± 0.0272	0.2811 ± 0.0068	0.415	29.05 [15]

In [15], the cumulative brainstem dose is reported as 12.2 Gy, which makes this sampling range infeasible. Therefore, we used a cumulative brainstem dose of 21 Gy, consistent with clinically acceptable limits [37], to ensure feasibility. For the brainstem, we report the dose to 0.03 cc (D0.03cc), while mean dose values are used for the remaining OARs; the reported dose values are based on the treatment plans in Parsai et al. [15].

Given that the spinal cord and brainstem are critical serial OARs that typically receive the highest priority in head and neck radiotherapy planning [38], higher weights ($w^{m}$ = 2 for spinal cord and brainstem, $w^{m}$ = 1.5 for esophagus) were assigned to these structures in the model to reflect their clinical importance in organ preservation (Supplementary Material G).

### 2.3 Performance metrics

To compare a standard IMRT plan with our optimized TFRT plan using variable feathering, we evaluated 1) end-of-treatment recovery and 2) dose to each OAR. For reference, we also included the standard TFRT [13], which schedules each OAR on a predetermined day with a one-week recovery between high-dose days.

## 3 Results

**Fig. 1:**
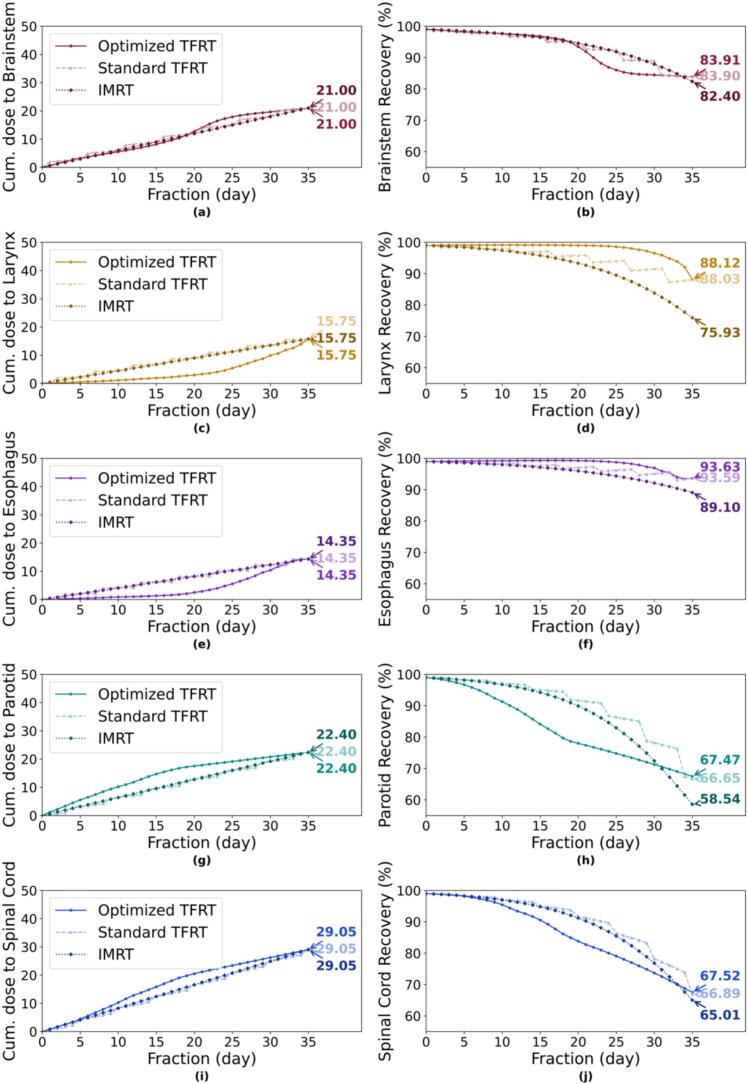
Comparison of Intensity Modulated Radiation Therapy (IMRT), standard Temporally Feathered Radiation Therapy (TFRT), and optimized dose-matched TFRT. (a) cumulative dose delivered to the brainstem (b) recovery of the brainstem (c) cumulative dose delivered to the larynx (d) recovery of the larynx (e) cumulative dose delivered to the esophagus (f) recovery of the esophagus (g) cumulative dose delivered to the parotid (h) recovery of the parotid (i) cumulative dose delivered to the spinal cord (j) recovery of the spinal cord.

For dose-matched (dose-restricted) case, the brainstem showed slightly higher recovery under optimized and standard TFRT plans (83.9%, SD = 0.21% for both) compared to the IMRT plan (82.4%), although cumulative doses were similar for all OARs (Figs. 1a–1b, Table 3). Receiving the same cumulative dose, the larynx demonstrated substantially better recovery (88.1%, SD = 0.77%) than the IMRT plan (75.9%) (Figs. 1c–1d, Table 3). The esophagus, feathered seven times, achieved 93.6% recovery (SD = 0.28%), matching the standard TFRT plan (SD = 0.28%) but exceeding the IMRT plan (89.1%) (Figs. 1e–1f, Table 3). The parotid received 22.4 Gy and showed improved recovery (67.5%, SD = 0.79%) compared to the standard TFRT plan (66.7%, SD = 0.83%) and the IMRT plan (58.5%) (Figs. 1g–1h, Table 3). The spinal cord, receiving a cumulative dose identical to the IMRT plan (∼29 Gy), exhibited recovery levels of 67.5% (SD = 0.31%), 66.9% (SD = 0.25%), and 65.0% under the optimized TFRT, standard TFRT, and IMRT plans, respectively (Figs. 1i–1j, Table 3).

**Table 3:** Comparison of cumulative dose, final recovery level, and feathering frequency for the optimized TFRT, standard TFRT, and IMRT plans under restricted (dose-matched) and unrestricted settings. Values are reported as mean ± standard deviation over 100 Monte Carlo simulations. TFRT: temporally feathered radiation therapy; IMRT: intensity modulated radiation therapy; OAR: organs at risk; SD: standard deviation.

OAR	Case	Method	Total Dose (Gy) Mean ± SD	Final Recovery (%) Mean ± SD	Range for # Times Feathered
Brainstem	Unrestricted	Optimized TFRT	19.36 ± 0.07	88.49 ± 0.31	6
		Standard TFRT	21.00 ± 0.00	83.90 ± 0.21	7
		IMRT	21.00 ± 0.00	82.40 ± 0.00	–
	Restricted	Optimized TFRT	21.00 ± 0.00	83.91 ± 0.21	7
		Standard TFRT	21.00 ± 0.00	83.90 ± 0.21	7
		IMRT	21.00 ± 0.00	82.40 ± 0.00	–
Larynx	Unrestricted	Optimized TFRT	17.57 ± 0.07	81.74 ± 0.87	8
		Standard TFRT	15.75 ± 0.00	88.03 ± 0.76	7
		IMRT	15.75 ± 0.00	75.93 ± 0.00	–
	Restricted	Optimized TFRT	15.75 ± 0.00	88.12 ± 0.77	7
		Standard TFRT	15.75 ± 0.00	88.03 ± 0.76	7
		IMRT	15.75 ± 0.00	75.93 ± 0.00	–
Esophagus	Unrestricted	Optimized TFRT	18.06 ± 0.14	84.81 ± 0.26	9
		Standard TFRT	14.35 ± 0.00	93.59 ± 0.28	7
		IMRT	14.35 ± 0.00	89.10 ± 0.00	–
	Restricted	Optimized TFRT	14.35 ± 0.00	93.63 ± 0.28	7
		Standard TFRT	14.35 ± 0.00	93.59 ± 0.28	7
		IMRT	14.35 ± 0.00	89.10 ± 0.00	–


OAR	Case	Method	Total Dose (Gy) Mean ± SD	Final Recovery (%) Mean ± SD	Range for # Times Feathered
Parotid	Unrestricted	Optimized TFRT	22.43 ± 0.20	67.18 ± 1.57	[7], [8]
		Standard TFRT	22.40 ± 0.00	66.65 ± 0.83	7
		IMRT	22.40 ± 0.00	58.54 ± 0.00	–
	Restricted	Optimized TFRT	22.40 ± 0.00	67.47 ± 0.79	7
		Standard TFRT	22.40 ± 0.00	66.65 ± 0.83	7
		IMRT	22.40 ± 0.00	58.54 ± 0.00	–
Spinal Cord	Unrestricted	Optimized TFRT	26.34 ± 0.26	77.86 ± 0.93	[4], [5]
		Standard TFRT	29.05 ± 0.00	66.89 ± 0.25	7
		IMRT	29.05 ± 0.00	65.01 ± 0.00	–
	Restricted	Optimized TFRT	29.05 ± 0.00	67.52 ± 0.31	7
		Standard TFRT	29.05 ± 0.00	66.89 ± 0.25	7
		IMRT	29.05 ± 0.00	65.01 ± 0.00	–

**Fig. 2:**
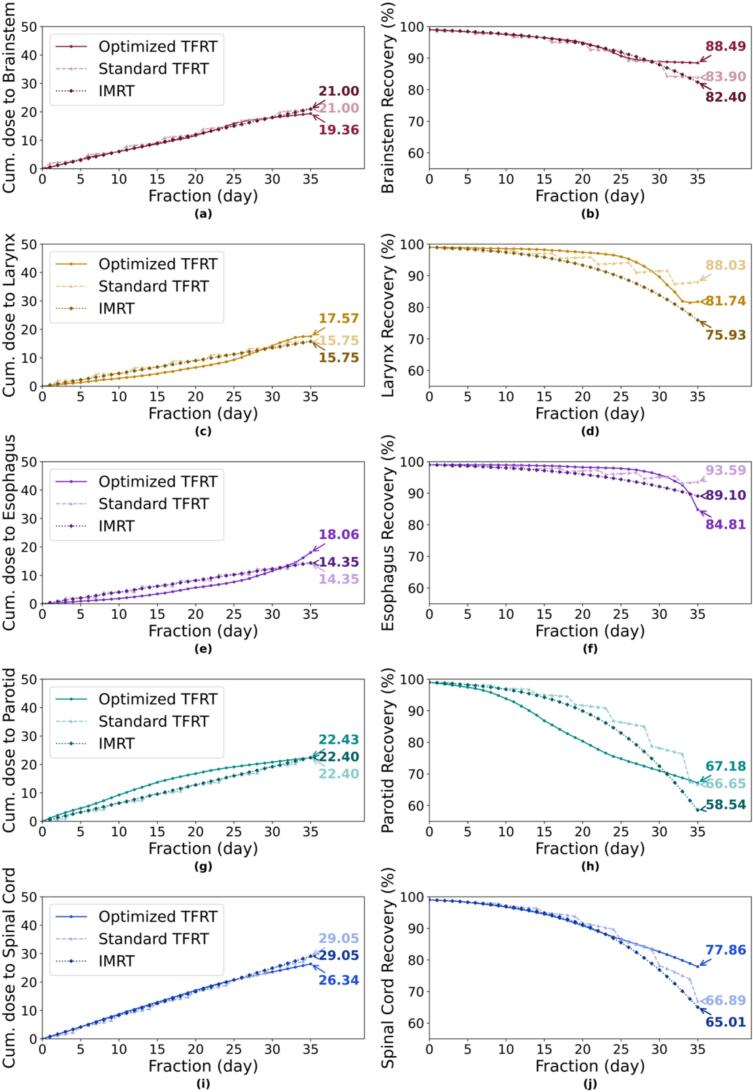
Comparison of Intensity Modulated Radiation Therapy (IMRT), standard Temporally Feathered Radiation Therapy (TFRT), and optimized dose-unrestricted TFRT when the brainstem, spinal cord and esophagus are prioritized compared to other organs-at-risk. (a) cumulative dose delivered to the brainstem (b) recovery of the brainstem (c) cumulative dose delivered to the larynx (d) recovery of the larynx (e) cumulative dose delivered to the esophagus (f) recovery of the esophagus (g) cumulative dose delivered to the parotid (h) recovery of the parotid (i) cumulative dose delivered to the spinal cord (j) recovery of the spinal cord.

For the dose-unrestricted case, the brainstem was feathered six times, which is fewer than the seven fractions used in the standard TFRT plan, reaching 88.5% recovery (SD = 0.31%) (Table 3). The esophagus was feathered nine times, with recovery of 84.8% (SD = 0.26%), while the larynx and parotid were feathered eight and seven to eight times, respectively (Table 3). The spinal cord was feathered four to five times on average (Table 3). The recovery outcomes demonstrated the advantages of the optimized TFRT approach. As shown in Figs. 2a–2b, the brainstem received a lower cumulative dose on average under the optimized TFRT plan compared to the IMRT and standard TFRT plans and achieved 88.5% mean recovery (SD = 0.31%) versus 82.4% for the IMRT plan and 83.9% (SD = 0.21%) for the standard TFRT plan (Table 3). The larynx, which was feathered eight times, received a higher mean cumulative dose (∼17.5 Gy, SD = 0.07 Gy) and achieved better recovery (81.7%, SD = 0.87%) compared to the IMRT plan (75.9%) but worse than the standard TFRT plan (88.0%, SD = 0.76%) (Figs. 2c–2d, Table 3). The esophagus, however, was feathered more frequently in our model. According to Figs. 2e–2f, the esophagus received a substantially higher mean cumulative dose (∼18.1 Gy, SD = 0.14 Gy) and exhibited reduced mean recovery (84.8%, SD = 0.26%) compared to the IMRT plan (89.1%) and the standard TFRT plan (93.6%, SD = 0.28%) (Table 3). The parotid was feathered seven times as in the standard TFRT plan and achieved 67.2% mean recovery (SD = 1.57%) at the end of treatment, which was substantially higher than the IMRT plan (58.5%) and slightly higher than the standard TFRT plan (66.7%, SD = 0.83%) (Figs. 2g–2h, Table 3). The spinal cord, feathered five times on average, received ∼26.3 Gy (SD = 0.26 Gy) and achieved superior recovery (77.9%, SD = 0.93%) compared to the standard TFRT plan (66.9%, SD = 0.25%) and the IMRT plan (65.0%) (Figs. 2i–2j, Table 3).

## 4 Discussion

We introduced a mathematical framework for optimizing TFRT feathering schedules based on heterogeneity in OAR radiosensitivity profiles. By incorporating OAR-specific radiosensitivity parameters, the model guided daily subplan selection to improve end-of-treatment OAR recovery. Overall, the findings indicated that temporal dose modulation, guided by the recovery levels, can enhance organ preservation while maintaining clinically relevant dose constraints.

Specifically, under dose-unrestricted conditions, optimized TFRT improved recovery for several critical structures, including the brainstem, spinal cord, and larynx, compared with standard TFRT and IMRT. Under dose-matched conditions, the model maintained comparable or superior recovery while constraining cumulative OAR dose to IMRT-equivalent levels, with notable improvements for the larynx, parotid, and esophagus. The optimized schedules adapted high-dose days over the treatment course while respecting recovery thresholds. We showed that that tailoring feathering schedules to OAR radiosensitivity can enhance TFRT outcomes even when cumulative dose is constrained. Clinical implementation of TFRT can follow established delivery approaches [15], [16].

The original TFRT study [13] investigated a simple nonuniform spatiotemporal fractionation at the OAR level with fixed 7-day rest time, while maintaining uniform dose to the tumor. Our model extended this concept by allowing more complex spatiotemporal alteration of the dose to the OARs. It leveraged the LQ model within a dynamical NTCP model to guide the treatment in reducing side effects through, for example, early feathering of OARs with lower α/β ratios, providing them with more inter-fraction recovery time.

The biological heterogeneity in the tumor cells and across various OARs pose a significant challenge to the well-accepted biological assumptions on the effects of radiation therapy, leading to suboptimal oncological outcomes and adverse events [39]. The OARs surrounding the tumor often react differently to radiation due to the variations in cellular repair mechanisms and each OAR's intrinsic radiosensitivity. As indicated in this study, this complexity can allow for more tailored radiation delivery by considering each organ's unique radiosensitivity and recovery dynamics, potentially preserving the function of critical organs and improving patient's quality of life.

The original TFRT study [13] indicated its benefits as a function of the dose delivered to the OARs on their high- and low-dose days. By incorporating of OAR's radiosensitivity differences, our model can broaden the pool of patients who may benefit from TFRT and provide a practical framework for patient selection in future randomized clinical trials, which is the gold standard for establishing its full potential in reducing OAR side effects and preservation. The optimized TFRT model can also tailor feathering schedules to prioritize specific OAR according to clinical needs. For instance, in patients with xerostomia, clinicians may prefer plans that further reduce the side effects to parotid beyond what IMRT achieves. Our model can accommodate such priorities by adjusting the weights assigned to OAR recovery, thereby enhancing personalized treatment planning.

As explored in ART framework, shifting from uniform fractionation to delivering variable daily doses can improve tumor control and/or reduce side effects, particularly in treatments with frequent fractionation [40]. Our model aligns closely with ART's objectives by utilizing the variable daily dose to HNC OARs to enhance organ sparing, thereby reducing patient side effects. Future research could explore incorporating daily on-treatment imaging data to further personalize the variable daily dose and optimize OARs recovery time.

Our model has limitations. The optimized TFRT relies on adjusted values of radiosensitivity parameters, similar in scale to those of the original TFRT study [13]. We acknowledge that the predicted end-of-treatment recovery values are sensitive to the assumed radiosensitivity parameters in addition to the inter-patient variability (which we have captured through Monte Carlo simulation). Reported radiosensitivity estimates in the literature exhibit substantial heterogeneity [19], [41], [42], reflecting differences in experimental conditions, modeling assumptions, and patient-specific variability. While our analysis could benefit from considering the inherent uncertainties in reported radiosensitivity parameters, insufficient data prevented us from conducting such analysis. Consequently, our current analysis should be interpreted within the context of these parameter uncertainties. Similar concern may arise for other NTCP model parameters, such as the recovery rate and the recovery threshold. Additionally, to illustrate proof of concept, we modeled OAR dose using sparing factors rather than full dose–volume optimization as performed in IMRT planning. Here, OAR dose represents the total daily dose inferred from the target dose, rather than a three-dimensional distribution. Although this commonly used approach [32], [33] isolates the temporal optimization component of TFRT, it does not capture spatial heterogeneity, organ-specific volume effects, or endpoint-dependent dose metrics (e.g., mean or maximum dose). Accordingly, the fractional OAR dose variations should be interpreted within this simplified framework and may not fully reflect isocurative clinical planning constraints. Future work should integrate both temporal and spatial optimization under clinically realistic deliverability conditions.

Our study indicated the potential of an improved end-of-the-treatment recovery for OARs of HNC when TFRT is further optimized for non-identical radiosensitivity of OARs, thus warranting further investigation in future radiation treatment planning for HNC.

## CRediT authorship contribution statement

**Aysenur Karagoz:** Writing – review & editing, Writing – original draft, Visualization, Validation, Software, Methodology, Investigation, Formal analysis, Data curation, Conceptualization. **Mehdi Hemmati:** Writing – review & editing, Writing – original draft, Visualization, Validation, Supervision, Software, Methodology, Investigation, Formal analysis, Data curation, Conceptualization. **Fatemeh Nosrat:** Writing – review & editing, Investigation, Data curation. **Panayiotis Mavroidis:** Writing – review & editing, Investigation, Data curation. **Cem Dede:** Writing – review & editing, Investigation, Data curation. **Lucas B. McCullum:** Writing – review & editing, Investigation, Data curation. **Raul Garcia:** Writing – review & editing, Investigation, Data curation. **Seyedmohammadhossein Hosseinian:** Writing – review & editing, Investigation, Formal analysis. **Jacob G. Scott:** Writing – review & editing, Methodology, Investigation. **James E. Bates:** Writing – review & editing. **Heiko Enderling:** Writing – review & editing, Investigation, Data curation. **Abdallah S.R. Mohamed:** Writing – review & editing. **Kristy K. Brock:** Writing – review & editing. **Andrew J. Schaefer:** Writing – review & editing, Supervision, Resources, Project administration, Methodology, Funding acquisition, Formal analysis, Conceptualization. **Clifton D. Fuller:** Writing – review & editing, Supervision, Resources, Project administration, Funding acquisition, Formal analysis, Conceptualization.

## Declaration of competing interest

The authors declare the following financial interests/personal relationships which may be considered as potential competing interests: (**Clifton D. Fuller** and **Andrew J. Schaefer** have received related grant funding and program support from the National Science Foundation (NSF)/National Institutes of Health (NIH) National Cancer Institute (NCI) Smart and Connected Health (SCH) Program (R01CA257814). **Clifton D. Fuller** has received grant funding from NIH National Institute of Dental and Craniofacial Research (NIDCR) Academic-Industrial Partnership (R01DE028290), and NIH NCI MD Anderson Cancer Center Support Grant (CCSG) Image-Driven Biologically-informed (IDBT) Program (P30CA016672). **Clifton D. Fuller** has additionally received related travel, honoraria, and/or registration fee waivers from Elekta AB, with further unrelated travel, honoraria, and/or registration fee waivers from institutions such as the American Society for Radiation Oncology and the Radiological Society of North America. **Clifton D. Fuller** has also received direct industry grant support, in-kind contributions, and honoraria from Elekta AB and collaborated in an unrelated consulting capacity with Varian/Siemens Healthineers, Philips Medical Systems, and GE Healthcare. **Kristy K. Brock** has received support from the NIH NCI through the Image Guided Cancer Therapy Research Program at MD Anderson Cancer Center (P30CA016672) and through a licensing agreement with RaySearch Laboratories AB. **Lucas B. McCullum** was supported by the National Institutes of Health (NIH) National Cancer Institute (NCI) Supplement program under R01CA257814-02S2. **Raul Garcia** was supposed by the NIH/NCI Supplement program under R01CA257814-02S1.)
