# Supplementary material for: Variable-interval temporal feathering to optimize organs-at-risk repair for head and neck adaptive radiotherapy
Source: Phys Imaging Radiat Oncol. 2026 Jun 19;40:101026. doi: 10.1016/j.phro.2026.101026 (PMC13471276; doi:10.1016/j.phro.2026.101026)
Supplement: Supplementary file 1 [file mmc1.pdf]

Supplementary Material A: The linear-quadratic model

The linear-quadratic (LQ) model [1] predicts the cell survival likelihood as a function of radiation dose d (Gy), given by

$$S(d) = e^{-\alpha d - \beta d^2}, \qquad (1)$$

where α (Gy^{-1}) and β (Gy^{-2}) are tissue-specific radiosensitivity parameters.

For n fractions each delivering d Gy, the biologically effective dose (BED) [2] is defined as $-ln(S)/\alpha$, where S is defined according to the LQ model (1), i.e.,

$$BED = nd\left(1 + \frac{d}{\alpha/\beta}\right). \qquad (2)$$

When the daily dose received by an OAR is nonuniform across treatment days (as is the case in TFRT treatment planning), the BED equation (2) becomes

$$BED = \sum_{t=1}^{n} d_t + \left(\frac{\beta}{\alpha}\right) \sum_{t=1}^{n} (d_t)^2, \qquad (3)$$

where d_t is the dose delivered to OAR in fraction $t = 1, \ldots, n$.

Supplementary Material B: Cells recovery from radiation-induced damage

We consider the nonspatial dynamical model of tissue response to radiation by Hanin and Zaider [3], which was originally used to evaluate the benefits of TFRT in reducing the side effects to OARs [4]. We let $0 \leq N(t) \leq 1$ represent the level of normal tissue recovery (with small values of $N(t)$ indicating severe damage). Then, the cell recovery process can be represented by the following ordinary differential equation (ODE):

$$\frac{dN(t)}{dt} = \mu N(t)(1 - N(t)) - RT(d)N(t)(1 - N(t)), \quad (4)$$

where μ (t^{-1}) is the organ-specific parameter representing the recovery rate and $RT(d)$ represents the likelihood of damaging each cell of the OAR receiving a radiation dose d. Using the LQ model (1), $RT(d) = 1 - S(d)$, i.e., $RT(d)$ represents the damaged fraction of the OAR when receiving a (single-fraction) radiation dose d.

Mathematical optimization models that incorporate ODE similar to (4) often present computational challenges. To approximate (4), Runge-Kutta (RK) methods [5] can be used. Here, we utilize the (forward) Euler's method, which is the first-order RK method. We approximate the ODE (4) by a set of difference equations, as follows:

$$N_{t+1} = N_t + \mu N_t(1 - N_t) - RT(d)N_t(1 - N_t),\ t = 1, \ldots, n - 1. \quad (5)$$

Our optimization model directly utilizes Equation (5) as a measure to guide decisions on OAR feathering in TFRT treatment planning.

Supplementary Material C: Constraints on daily subplan selection

If the sparing factor of OAR m in the treatment subplan for fraction t is denoted by $f_t^m \in [0,1]$, then the dose delivered to the OAR in fraction t is computed as $f_t^m d$, where d is the daily (fixed) dose delivered to the tumor. Accordingly, each treatment subplan comprises Msparing factors, one corresponding to each OAR. By varying an OAR's sparing factors across treatment days, we allow a nonuniform daily dose to be received by the OAR.

For each subplan $k = 1,\ldots,M$, we denote by p^k an M-dimensional vector, comprising individual OAR's sparing factors $p^{k,m} \in [0,1], m = 1,\ldots,M$. Accordingly, we define binary decision variables $y_t^m, m = 1,\ldots,M, t = 1,\ldots,n$, taking the value of one if the subplan corresponding to the designated OAR m is administered in fraction t. We enforce

$$\sum_{m=1}^{M} y_t^m = 1, t = 1,\ldots,n, \quad (6)$$

and

$$f_t^m = \sum_{k=1}^{M} p^{k,m} y_t^m, m = 1,\ldots,M, t = 1,\ldots,n. \quad (7)$$

Equation (6) implies that only one subplan is selected for each weekday, indicating exactly one OAR is feathered. The definition of sparing factor of an OAR in any fraction is enforced through Equation (7). Since the daily dose to the tumor is 2 Gy, we enforce

$$d_t^m = 2(f_t^m), m = 1,\ldots,M, t = 1,\ldots,n. \quad (8)$$

Supplementary Material D: Constraints on maximum BED received by the OARs

We let the parameters $\tau_m, m = 1, \ldots, M$ be the maximum allowable BED received by the OAR m by the end of the treatment. Given $m = 1, \ldots, M$ and $t = 1, \ldots, n$, we define the decision variable d_t^m as the dose received by OAR m in fraction t, and enforce the following constraint

$$\sum_{t=1}^{n} d_t^m + \left(\frac{\beta}{\alpha}\right) \sum_{t=1}^{n} (d_t^m)^2 \leq \tau_m, m = 1, \ldots, M. \quad (9)$$

Supplementary Material E: Constraints on OAR feathering based on its recovery

The recovery of an OAR m is computed as in Equation (5), i.e.,

$$N_{t+1}^{m} = N_t^m + \mu^m N_t^m (1 - N_t^m) \; - \; RT(d_t^m) N_t^m (1 - N_t^m) \,, \; t = 1, \ldots, n-1. \qquad (10)$$

Similar to the original study of TFRT, we assume that the OAR is at tissue homeostasis before radiation with a 1% turnover rate [13], hence we enforce the initial following initial condition

$$N_1^m = 0.99, \; m = 1, \ldots, M. \qquad (11)$$

We will leverage the recovery level of each OAR to guide our choice of the subplan to be administered each weekday, i.e., the value of the decision variables $y_t^m, m = 1, \ldots, M, t = 1, \ldots, n$. We define ξ^m as the threshold for the minimum recovery level required for OAR $m = 1, \ldots, M$ before it can receive a dose higher than the daily standard IMRT dose. We enforce

$$y_t^m \leq N_t^m \; - \; \xi^m \; + 1 \,, m = 1, \ldots, M, t = 1, \ldots, n. \qquad (12)$$

Supplementary Material F: Constraints on identical total dose delivery under IMRT

As reported in the study by Alfonso et al. [13], the total cumulative dose received by OARs under TFRT may be slightly more than IMRT. Hence, we consider two variations of our model depending on whether the total dose received by OARs under TFRT is at least the total dose delivered under IMRT. To enforce this constraint, we define $D_{IMRT}^{m}, m = 1, \dots, M$ be the total dose received by each OAR in standard IMRT. We impose

$$\sum_{t=1}^{n} d_t^m \geq D_{IMRT}^{m}, m = 1, \dots, M, \quad (13)$$

Supplementary Material G: Integrated Model

We define w^m as the weight (indicating the importance) of the OAR $m = 1, \dots, M$. Our model is presented as follows

$$max \quad \sum_{m=1}^{M} w^m N_n^m \tag{14a}$$

$$\text{Constraints (6)-(12)} \tag{14b}$$

$$y_t^m \in \{0,1\}, \quad m = 1, \dots, M, t = 1, \dots, n \tag{14c}$$

$$N_t^m \leq 1, \quad m = 1, \dots, M, t = 1, \dots, n \tag{14d}$$

The proposed model (14) seeks the maximum weighted OARs recovery at the end of the treatment subject to the constraints introduced earlier in this section. Constraints (14c) imply the definition of the y decision variables and constraints (14d) enforce the logical upper bound on the recovery of each OAR.

References

[1] Brenner DJ. The Linear-Quadratic Model Is an Appropriate Methodology for Determining Isoeffective Doses at Large Doses Per Fraction. Semin Radiat Oncol 2008;18:234–9. https://doi.org/10.1016/j.semradonc.2008.04.004.

[2] Fowler JF. 21 years of biologically effective dose. Br J Radiol 2010;83:554–68. https://doi.org/10.1259/bjr/31372149.

[3] Hanin L, Zaider M. A mechanistic description of radiation-induced damage to normal tissue and its healing kinetics. Phys Med Biol 2013;58:825–39. https://doi.org/10.1088/0031-9155/58/4/825.

[4] López Alfonso JC, Parsai S, Joshi N, Godley A, Shah C, Koyfman SA, et al. Temporally feathered intensity-modulated radiation therapy: A planning technique to reduce normal tissue toxicity. Med Phys 2018;45:3466–74. https://doi.org/10.1002/mp.12988.

[5] Burden RL, Faires JD, Burden AM. Numerical analysis. Tenth edition. Boston, MA: Cengage Learning; 2016.
